# The impact of CP12 on the metabolome of cyanobacteria under fluctuating CO_2_ conditions

**DOI:** 10.3389/fpls.2025.1674721

**Published:** 2025-09-23

**Authors:** Stefan Lucius, Stéphanie Arrivault, Regina Feil, Luna Alvarenga-Lucius, Martin Hagemann

**Affiliations:** ^1^ Plant Physiology, Institute of Biosciences, University of Rostock, Rostock, Germany; ^2^ Max Planck Institute of Molecular Plant Physiology, Potsdam-Golm, Germany; ^3^ Interdisciplinary Faculty, Department Life, Light and Matter, University of Rostock, Rostock, Germany

**Keywords:** carbon metabolism, cellular concentrations, glycolysis, inorganic carbon, mutant, redox regulation

## Abstract

All organisms that perform oxygenic photosynthesis fix inorganic CO_2_ through the Calvin-Benson-Bassham (CBB) cycle, which is then converted into many organic compounds in associated pathways of primary carbon and nitrogen metabolism. Autotrophic CO_2_ fixation is only possible in the light, while under dark conditions, phototrophs adopt a heterotrophic lifestyle using stored organic carbon reserves. The switch between autotrophic and heterotrophic lifestyles often involves the activation and inactivation of key enzymes by redox regulation, including the regulatory protein CP12. In the present study, we analyzed the primary metabolism of the model cyanobacterium *Synechocystis* sp. PCC 6803 under different CO_2_ conditions in continuous light using targeted metabolomics. The comparison of wild type and a mutant with deleted CP12 showed that this regulatory protein is crucial for the acclimation of the metabolism when shifted for 1 h or 3 h from high to low CO_2_. Especially 1 h after shift from high into low CO_2_, many metabolites of the primary carbon and nitrogen metabolism showed a strong transient increase in the mutant Δ*cp12*. Moreover, distinct differences were also observed when the strains were grown for longer times at high or low CO_2_ conditions. Collectively, our results show that the absence of CP12 not only affected the CBB cycle under diurnal conditions but also had a marked impact on glycogen catabolism and associated nitrogen metabolism in cyanobacteria exposed to different CO_2_ conditions in continuous light.

## Introduction

Organisms performing oxygenic photosynthesis, such as cyanobacteria, algae and plants, fix inorganic CO_2_ in the Calvin-Benson-Bassham (CBB) cycle to synthesize organic carbon. The CO_2_ fixation in the CBB cycle is catalyzed by the enzyme ribulose 1,5-bisphosphate carboxylase/oxygenase (RubisCO), leading to the synthesis of two 3-phosphoglycerate (3PGA) molecules by the binding of CO_2_ on ribulose 1,5-bisphosphate (RuBP). However, RubisCO proteins of type 1, which are also found in cyanobacteria including our model *Synechocystis* sp. PCC 6803, are characterized by rather low CO_2_ affinity and specificity (Tcherkez et al., 2006). Under ambient CO_2_ conditions, the enzyme can also utilize O_2_ in its oxygenase reaction, resulting in the synthesis of one 3PGA and one 2-phosphoglycolate (2PG). The side product 2PG can inhibit several enzymes in the primary carbon metabolism of oxygenic phototrophs; therefore, it is salvaged in the process of photorespiration, which releases approximately one-fourth of the organic carbon as CO_2_ and requires cellular energy and reducing power (reviewed in Hagemann et al., 2013).

In contrast to land plants, organisms that perform oxygenic photosynthesis in aquatic habitats are faced with the low solubility of CO_2_ in water. CO_2_ dissolution primarily depends on water pH and temperature, resulting in fluctuating CO_2_ availability (Kupriyanova et al., 2023). To thrive under low and fluctuating CO_2_ conditions, cyanobacteria and many algal groups evolved efficient CO_2_-concentrating mechanisms (CCMs) that concentrate CO_2_ in the vicinity of RubisCO, thereby promoting its carboxylase activity and decreasing the extent of photorespiration (Raven et al., 2017). The cyanobacterial CCM involves several uptake systems for inorganic carbon (Ci) in the forms of bicarbonate and CO_2_, leading to the accumulation of bicarbonate inside the cell, which is then reconverted into CO_2_ by carbonic anhydrase inside the carboxysome, a prokaryotic compartment harboring RubisCO. The expression of CCM components is primarily regulated by varying Ci amounts at the transcriptional level (reviewed in Hagemann et al., 2021). It has also been demonstrated that fluctuating Ci conditions have a marked impact on the metabolome of the model cyanobacterium *Synechocystis* sp. PCC 6803 (hereafter *Synechocystis*) (e.g., Eisenhut et al., 2008; Hackenberg et al., 2012). However, transcript and protein levels of enzymes of the central carbon metabolism did not show significant changes under fluctuating Ci conditions (Klähn et al., 2015; Jahn et al., 2018; Barske et al., 2023).

In addition to sufficient Ci availability, photosynthetic CO_2_ fixation is also strictly dependent on light conditions, as the photosynthetic complexes convert light energy into ATP and NADPH, which are needed in the CBB cycle and other potential sinks (e.g., Grund et al., 2019). Therefore, oxygenic phototrophs such as cyanobacteria exhibit autotrophic metabolism only during the day. In the absence of light, i.e., during the night they perform a heterotrophic lifestyle by using stored carbohydrates such as glycogen in catabolic processes for energy production and metabolic purposes (reviewed in Lucius and Hagemann, 2024). The activation of photosynthesis and the CBB cycle after the onset of light is regulated on different levels; among them, redox regulation of enzyme activities is regarded as the most important process.

In contrast to plants, most enzymes of the CBB cycle in cyanobacteria are not directly redox-regulated to activate/inactivate CO_2_ fixation in diurnal light/dark cycles (e.g., Gurrieri et al., 2021). Instead, the small redox regulator protein CP12 has been shown to bind and inactivate glyceraldehyde-3-phosphate dehydrogenase (GapDH) and phosphoribulokinase (PRK) from the CBB cycle under oxidative conditions, i.e., after shifts from light into dark conditions (Blanc-Garin et al., 2022; Lucius et al., 2022). CP12 (chloroplast protein of 12 kDa) was first discovered in plants (Wedel and Soll, 1998). This small, intrinsically disordered protein has been identified in nearly all cyanobacteria, algae, and plants (reviewed in López-Calcagno et al., 2014; Gurrieri et al., 2021). Canonical CP12 proteins are characterized by two cysteine (Cys) pairs, one near the N-terminal and another near the C-terminal part that can form disulfide bonds, which permits CP12 to bind GapDH and PRK. Biochemical and structural analyses showed that first two GapDH tetramers and then two PRK dimers are recruited by four oxidized CP12 molecules (McFarlane et al., 2019; Yu et al., 2020). Recently, we could visualize the appearance and disappearance of CP12/GapDH/PRK complexes under oxidizing (switching off light) and reducing (switching on light) conditions, respectively, in *Synechocystis* using Yfp-labelled proteins (Lucius et al., 2022). Our previous study also provided hints that the absence of CP12 might have a broader impact on cyanobacterial metabolism. The activity of glucose-6-phosphate dehydrogenase (G6PDH), the entrance enzyme of the oxidative pentose-phosphate (OPP) pathway, was decreased, which affected the utilization of external glucose in dark phases in the Δ*cp12* mutant (Lucius et al., 2022). It remains unclear if CP12 has other specific targets in cyanobacteria in addition to GapDH and PRK. However, it has been reported that CP12 in *Chlamydomonas* can be associated with aldolase, which also plays a vital role in sugar metabolism in oxygenic phototrophs (Erales et al., 2008). Moreover, the *Synechocystis* CP12 protein was found to be differentially phosphorylated under varying Ci conditions (Spät et al., 2021), which points to a potential additional regulatory layer.

In the present study, we provide new data on the impact of CP12-mediated regulation on the photoautotrophic metabolism in cyanobacteria under different Ci conditions in continuous light. For this purpose, we compared the metabolome of the wild type (WT) and mutant Δ*cp12* of *Synechocystis* under ambient CO_2_ conditions (0.04% CO_2_, low CO_2_, LC) and CO_2_-enriched (5% CO_2_, high CO_2_, HC) conditions, respectively. For metabolite quantification, we combined two advanced methods, which enabled us to obtain a comprehensive picture of central carbon and nitrogen metabolism. All experiments were performed under continuous light to avoid the already well investigated redox-related CP12 effects under diurnal conditions. Our results show that the CP12-mediated regulation has a high importance for the rapid acclimation of the central metabolism in *Synechocystis* towards fluctuating CO_2_ under constant light conditions.

## Materials and methods

### Strains and cultivation

For all experiments, the glucose-tolerant wild-type (WT) strain of *Synechocystis* sp. PCC 6803 was used. The construction and characterization of the Δ*cp12* deletion mutant of *Synechocystis* was described previously (Lucius et al., 2022). Cells of *Synechocystis* WT and Δ*cp12* were pre-cultivated for one week in glass tubes by continuous sparking with air of either low CO_2_ (ambient air with 0.04% CO_2_, LC, results in approximately 0.6 mM Ci in the medium) or high CO_2_ (5%, HC, results in approximately 50 mM Ci in the medium) levels in buffered BG11 medium (Rippka et al., 1979; TES pH 8.0) with respective antibiotics (mutant Δ*cp12* with 50 µg ml^-1^ kanamycin) at continuous light of 100 µmol photons m^-2^ s^-1^ (warm light fluorescent tubes). Cells were then harvested by centrifugation and resuspended in fresh BG11 medium to an optical density at 750 nm (OD_750_) of 1.0 (approximately containing 10^9^ cells ml^-1^). OD_750_ of cell suspensions was measured after appropriate dilutions with a spectrophotometer (Cary 50, Varian, UK) using glass cuvettes of 1 cm path. After 24 h of acclimation, cultures were adjusted to an OD_750_ of 1.0 with fresh BG11. To initiate the experiment, these cultures were maintained for 3 h in their respective CO_2_ conditions. To shift cultures from HC to LC, cells were harvested by centrifugation and then re-suspended in an equal volume of fresh BG11 medium, after which they were connected to ambient air aeration.

### Sampling and metabolite analysis

**Figure 1:**
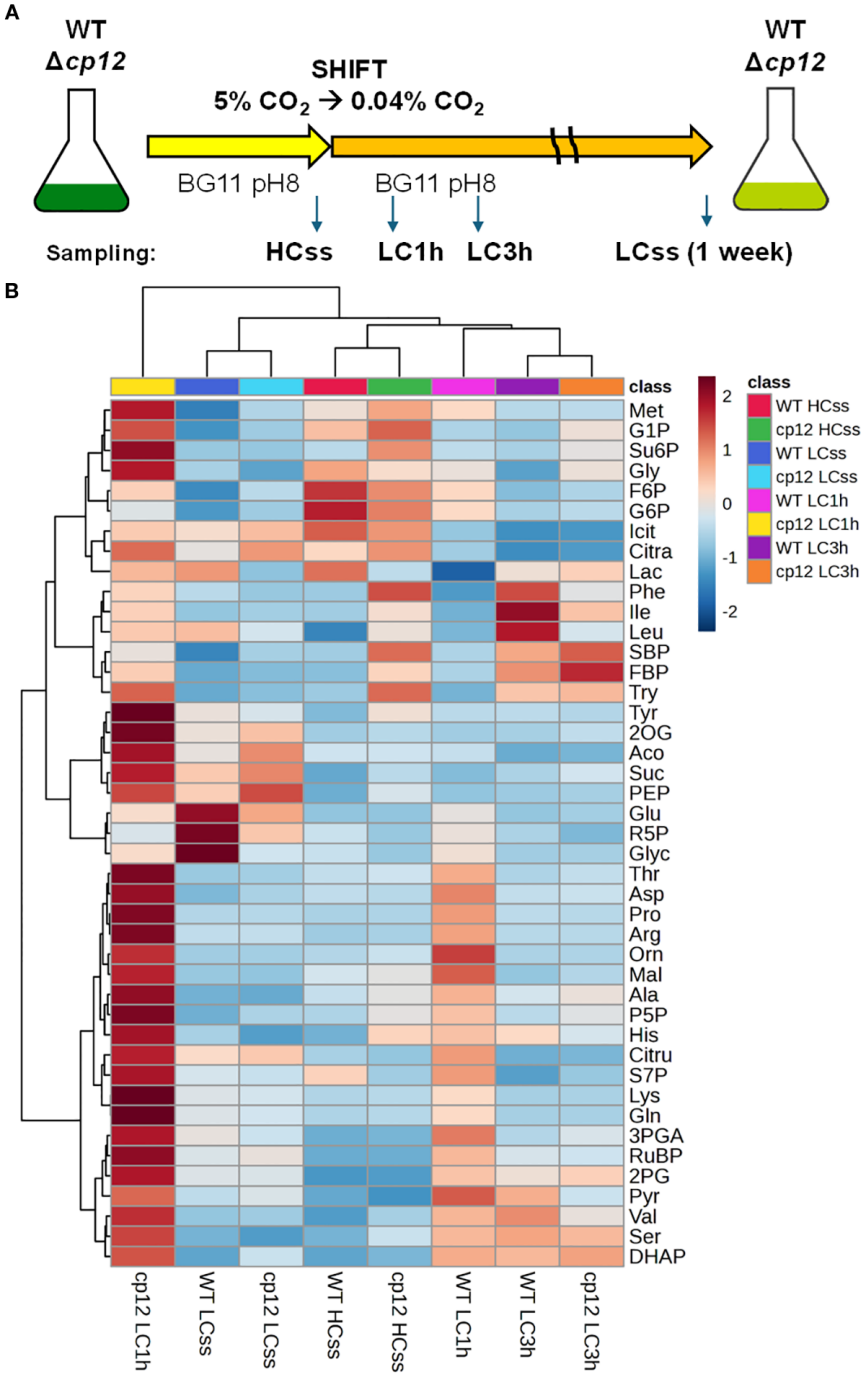
Experimental design and heat map of metabolic changes. **(A)** The upper panel shows the experimental design. Cells of the *Synechocystis* sp. PCC 6803 wild type (WT) and the mutant Δ*cp12* were grown for at least one week at 5% CO_2_ (HC) before sampling steady-state values (ss). Then, cells acclimated to HC were shifted to ambient, low CO_2_ (LC) conditions and were sampled after 1 or 3 h. In addition, LC-grown cells were sampled after one-week growth at ambient CO_2_ (LCss). The experiment was repeated with independent cultivations. In each experiment, four biological replicates were cultivated and used for sampling. **(B)** The lower panel displays a heat map showing relative changes of all metabolites at all sampling points in the two strains as log_2_ fold changes. The upper clusters group the different sampling times (all strains and sampling times are displayed below), whereas the clustering at the left side groups the different metabolites (all measured metabolites are displayed right: G6P, glucose 6-phosphate; F6P, fructose 6-phosphate; G1P glucose 1-phosphate; P5P, ribulose 5-phosphate + xylulose 5-phosphate; R5P ribose 5-phosphate; DHAP, dihydroxyacetone-phosphate; FBP, fructose 1,6-bisphosphate; S7P, sedoheptulose 7-phosphate; 2PG, 2-phosphoglycolate; SBP, sedoheptulose 1,7-bisphosphate; RuBP, ribulose 1,5-bisphosphate; Su6P, sucrose 6-phosphate; 3PGA, 3-phosphoglycerate; PEP, phosphoenolpyruvate; Glyc, glycerate; Mal, malate; Aco, aconitic acid; Citra, citric acid; Icit, isocitric acid; 2OG, 2-ketoglutaric acid; Lac, lactic acid; Suc, succinate; Pyr, pyruvate; Ala, Alanine; Arg, Arginine; Asp, Aspartic Acid; Glu, Glutamic Acid; Gln, Glutamine; Gly, Glycine; His, Histidine; Ile, Isoleucine; Leu, Leucine; Lys, Lysine; Met, Methionine; Phe, Phenylalanine; Pro, Proline; Ser, Serine; Thr, Threonine; Try, Tryptophan; Tyr, Tyrosine; Val, Valine; Orn, Ornithine; Cit, Citrulline.

Right before, as well as 1 h and 3 h after the HC to LC shifts, four samples of 8 ml were harvested from each independent culture vessel (each with 100 ml total cell suspension) and quenched directly in 16 ml 70% methanol cooled by dry ice (**Figure 1**). Quenched cells were pelleted by centrifugation (7 min, 10000 g, 4°C), supernatants were discarded, and cell pellets were quickly frozen in liquid nitrogen and stored at -80°C for up to one month until extraction. For metabolite extraction, quenched cell pellets were re-suspended in -20°C cold 47% methanol/chloroform (5:1, v/v), followed by four cycles of freezing suspensions in liquid nitrogen, 1 h storage in -80°C freezer and thawing on ice. Final extracts were lyophilized (Christ Alpha 2–4 lyophilizer, Christ, Germany), re-suspended in 250 µl MS-grade pure water and filtered (MultiScreen filter plate with Ultracel-10 membrane, Millipore).

The extracts were divided in two aliquots, which were then used for different metabolite quantification methods. Phosphorylated intermediates were quantified by liquid chromatography linked to tandem mass spectrometry (LC-MS/MS, Thermo-Fisher-Scientific, USA) using reverse phase LC and anion exchange LC as described previously in detail (Lunn et al., 2006; Arrivault et al., 2009; 2015). Most organic acids and amino acids were quantified using another LC-MS/MS system (LCMS-8050, Shimadzu, Japan) as has been described before in detail (Reinholdt et al., 2019). In the latter case, the compounds were identified and quantified using the multiple reaction monitoring (MRM) values given in the LC-MS/MS method package and the LabSolutions software package (Shimadzu, Japan). Losses during the extraction procedure were calculated by normalization of signal intensity to that of the internal standard carnitine, i.e. 30 µg of carnitine were added to each sample.

Cellular glycogen was determined according to Gründel et al. (2012) with modifications described by Lucius et al. (2021). During growth experiments, two 5 ml aliquots of cells were harvested from the cultures and pelleted by centrifugation. The pellet was resuspended in 300 µl 30% KOH (w/v), and glycogen was precipitated by 900 µl ice-cold pure ethanol (Carl Roth, Germany). Washed and dry pellets were resuspended in 200 µl sodium acetate buffer (100 mM, pH 4.5) containing 21 U of amyloglucosidase (Sigma-Aldrich, Germany). Glucose in supernatants was determined using the o-toluidine reagent (Carl Roth, Germany).

### Data evaluation and statistical analysis

The experiments were repeated twice with independent cultivations. In each experiment, four biological replicates were cultivated and used for sampling. Data evaluation for LC-MS/MS was done by calculating the averages of absolute metabolite contents per sampling point within each biological replicate. Absolute quantification was done by the evaluation of analyses of internal standards of authentic substances with known amounts for each quantified metabolite in the same runs. Final metabolite values were obtained by calculating the average and standard deviations of all biological replicates, which were normalized to the sampling volume and total biomass measured as optical density at 750 nm (OD_750_; the data set is given in **Supplementary Table S1**). Cellular concentrations were estimated by converting our measured data per biomass as optical density (OD) of 750 nm into single cell volume (mm^3^). This conversion was done using a calibration curve of *Synechocystis* suspension diluted at different OD_750_ values and measured total cell volume mm^3^ ml^-1^ by a Coulter counter in each diluted suspension to normalize metabolite levels to cell volume. The following equation was used: biovolume [mm^3^ ml^-1^] = OD_750_ x 0.919.

The glycogen datasets are representative of at least three biological replicates. A Student’s t-test with a significance level of 5% (p ≤ 0.05) was performed to detect specific differences for metabolite and glycogen data (see **Supplementary Table S1**). All t-tests were performed with six repetitions, so the data were homogeneous. A significant deviation of every single data point of a mutant strain compared to the respective WT data is marked by an asterisk. The Metaboanalyst 6.0 platform (Ewald et al., 2024) was used to analyze and display the metabolic data, as a heat map including the cluster analysis, principal component analyses (PCAs) and self-organizing mapping analysis (SOM). These analyses included total metabolite data from all replicates. The PCA analyzed the distribution of the 10 main metabolites in the samples and separated the treatments/strains accordingly.

## Results

### Global changes

In order to investigate the impact of CP12-mediated CBB cycle regulation under fluctuating Ci conditions, we compared the metabolome of *Synechocystis* WT cells and mutant Δ*cp12* under different CO_2_ regimes at constant light to rule out light/dark related redox changes (**Figure 1A**). The cells were either long-term acclimated to HC or LC conditions to determine the metabolite composition under steady-state conditions (HCss and LCss, respectively). Furthermore, metabolite transients were investigated in cells shifted from HC to LC for 1 h or 3 h (LC1h and LC3h, respectively). The extracts were analyzed by two targeted LC-MS/MS analyses, permitting the quantification of more than 40 metabolites of the primary C- and N-metabolism (**Supplementary Table S1**). The measured concentrations per OD_750_ and ml algal suspension were used to calculate cellular metabolite concentrations. This was done using a calibration curve between different OD_750_ values and total cell volume mm^3^ to normalize metabolite levels to cell volume. These data are displayed in **Supplementary Table S2** and will be helpful in future modelling approaches, in which known biochemical features of *Synechocystis* enzymes (i.e., k_m_ and v_max_ values) can be combined with metabolite amounts.

The first global analysis of the entire data set revealed that the metabolomes from steady-state HC- and LC-grown cells of the two strains clustered together, as was also observed for the samples from the two strains 3 h after the LC shift, despite differences in distinct intermediates. However, the metabolome of the mutant Δ*cp12* was found to differ from that of all other samples examined 1 h after the LC shift (**Figure 1**), when many of the detected metabolites exhibited increased values in mutant cells. This finding suggests that the CP12-mediated regulation buffers the CBB cycle, as well as the entire metabolism, against sudden changes, and its absence has a widespread impact on many intermediates of primary C- and N-metabolism. The PCA and SOM analyses (**Supplementary Figure S1**) exhibited comparable trends, with WT and Δ*cp12* at steady state LC (LCss) grouped in a single cluster (Cluster 0_0), and all other treatments, except Δ*cp12* LC1h, grouped in another (Cluster 0_1). The Cluster 0_2 was found to comprise solely of Δ*cp12* LC1h, which exhibited a distinct metabolism pattern (**Supplementary Figure S1B**).

Such a tendency was anticipated for Δ*cp12* cells when shifted from HC to LC, given the direct, redox-dependent interaction of CP12 with two pivotal enzymes from the CBB pathway. Alterations in the CBB cycle flux and carbon partitioning have been observed to exert considerable influence on other associated biochemical pathways (e.g., Stitt, 2013). Furthermore, the metabolite sucrose 6-phosphate exhibited the greatest significance between the clusters (**Supplementary Table S1**), which resembled the situation in tobacco plants showing elevated sugar accumulation after downregulation of CP12 (Howard et al., 2011). It is interesting to note that the formation of sucrose 6-phosphate begins with the CBB products, fructose 6-phosphate and UDP-glucose. This pathway acts as a carbon sink, helping to regulate the flow of fixed carbon when demand for growth or storage of glycogen is limited, thereby balancing NADPH and ATP usage and indirectly supporting redox homeostasis (e.g., Lin et al., 2020).

### CBB cycle

Because CP12 directly regulates the activities of two CBB enzymes in a redox-dependent manner, we expected the most prominent changes in metabolites of this cycle under fluctuating Ci conditions, even in continuous light. The LC-MS/MS methods permitted the quantification of 10 CBB cycle intermediates. Unfortunately, glyceraldehyde 3-phosphate (Gap) cannot be detected due to its instability and low levels; however, we assume that it is in equilibrium with the detected dihydroxyacetone-phosphate (DHAP) through the triosephosphate isomerase (TPI) activity. Furthermore, we have no reliable data for erythrose 4-phosphate. An 11th metabolite, 2PG, the first intermediate of photorespiration is also included with the CBB metabolites as it is also a product of RubisCO.

**Figure 2:**
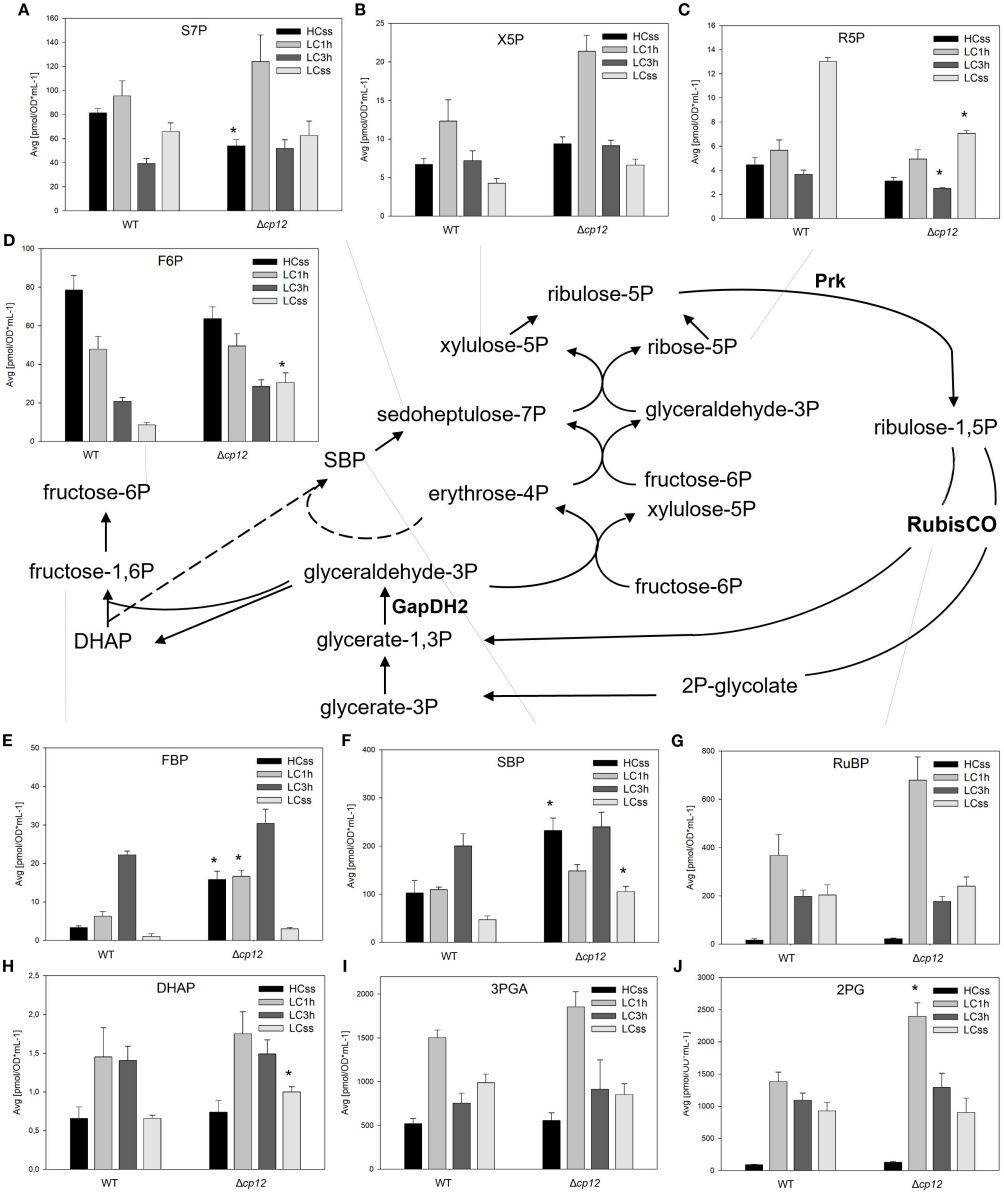
Schematic presentation of the CBB cycle and graphs showing the concentration of intermediates under different Ci conditions. Cells of the *Synechocystis* sp. PCC 6803 wild type (WT) and the mutant Δ*cp12* were grown at either 5% CO_2_ (HCss) or ambient 0.04% CO_2_ (LCss), and cells acclimated to HC were shifted to LC conditions (LC1h or LC3h). CBB cycle intermediates [panels **(A–J)**] were quantified using GC-MS and the values are displayed as pmol OD_750_^–1^ ml^-1^ (mean values from two independent experiments with each four biological replicates and standard deviations, statistically significant differences (p ≤ 0.05) between WT and mutant are marked with asterisks). Abbreviations and further details are explained in **Figure 1**. The two CP12-regulated enzymes GapDH2 and PRK are shown.

Regarding general changes under steady-state conditions, three groups of CBB intermediates could be distinguished consistently in the two strains. Increased levels of 2PG, 3PGA, ribulose 1,5-bisphosphate (RuBP), and ribose 5-phosphate (R5P) were measured under LC compared to HC conditions (**Figures 2C, G, I, J**), whereas decreased amounts were detected for fructose 6-phosphate (F6P), fructose 1,6-bisphosphate (FBP), and sedoheptulose 1,7-bisphosphate (SBP) (**Figures 2D–F**). Almost no changes in the steady-state levels were detected for DHAP, sedoheptulose 7-bisphosphate (S7P), and xylulose 5-phosphate (X5P) in the two strains (**Figures 2A, B, H**). All CBB intermediates exhibited larger deviations in transition time after shifts from HC to LC. Most intermediates became elevated 1 h or 3 h after LC shift, only the F6P values showed a gradual decline from the high HC values towards to low LC values during the shift period (**Figure 2**). For many intermediates, a similar relative change was found in the mutant Δ*cp12* as in WT. However, a twofold higher transient accumulation of RuBP, 2PG, and X5P occurred in mutant cells compared to WT 1 h after shift to LC (**Figures 2B, G, J**). Furthermore, the steady-state values of FBP and SBP were more than twofold higher in the mutant than in WT cells under HC conditions (**Figures 2E, F**), while those of F6P and S7P appeared diminished under the same conditions (**Figures 2A, D**). The steady-state amounts of other CBB intermediates were almost similar in both strains after long-term LC acclimation (**Figure 2**).

### Glycogen and intermediates of glucose catabolism via glycolysis and the OPP pathway

**Figure 3:**
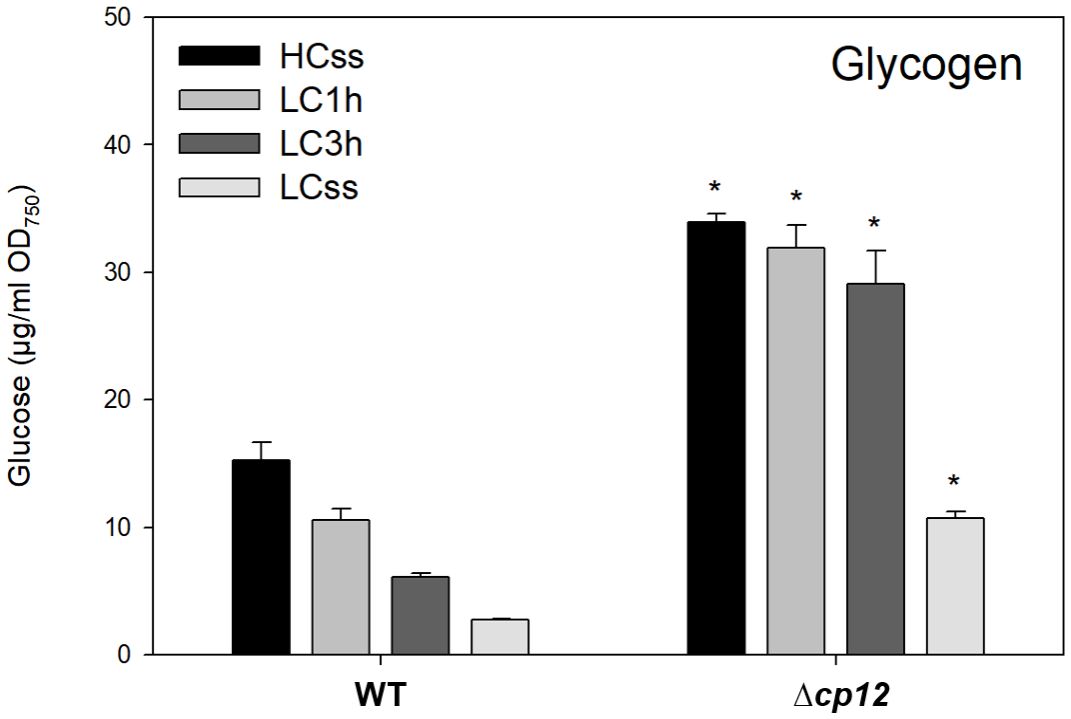
Glycogen levels under different Ci conditions. Cells of the *Synechocystis* sp. PCC 6803 wild type (WT) and the mutant Δ*cp12* were grown at either 5% CO_2_ (HCss) or ambient 0.04% CO_2_ (LCss), and cells acclimated to HC were shifted to LC conditions (LC1h or LC3h). Glycogen was quantified as soluble glucose after α-glucosidase treated cells (mean values from two independent experiments with each four biological replicates and standard deviations, statistically significant differences (p ≤ 0.05) between WT and mutant are marked with asterisks).

It has been observed before that excess CO_2_ at HC conditions stimulates glycogen accumulation in *Synechocystis*, which is consumed under LC conditions (e.g. Eisenhut et al., 2008). Accordingly, 5 times more glycogen was detected under steady-state conditions in HC-grown compared to LC-acclimated WT cells (**Figure 3**). The internal glycogen pool rapidly declined when WT cells were shifted from HC to LC conditions under continuous light (**Figure 3**). It has been shown that the glucose derived from glycogen breakdown can replenish the CBB cycle with organic carbon for RuBP synthesis under Ci-limiting conditions when the CCM is not fully induced (Makowka et al., 2020; Lucius et al., 2021). In the present study, the glycogen levels were entirely different in mutant Δ*cp12* compared to WT (**Figure 3**). As shown before (Lucius et al., 2022), the glycogen steady-state levels were more than twofold higher in mutant cells under HC as well as LC conditions. A particularly noteworthy finding was the slower decline in accumulated glycogen when mutant cells were transitioned from HC to LC. WT cells decreased twofold faster the glycogen pool after HC to LC shift, i.e., these cells consumed per hour between 4.5 to 3 µg glucose per ml and OD_750_ 1 and 3 h after the shift, respectively, whereas Δ*cp12* cells consumed per hour only 2 to 1.6 µg glucose per ml and OD_750_ 1 and 3 h after the shift. These results suggest that the increased glycogen levels in the absence of CP12 can be rather explained by a slower turnover of the glycogen pool, because especially its degradation seems to be slower as has been suggested before in experiments with glucose supplementation (Lucius et al., 2022).

**Figure 4:**
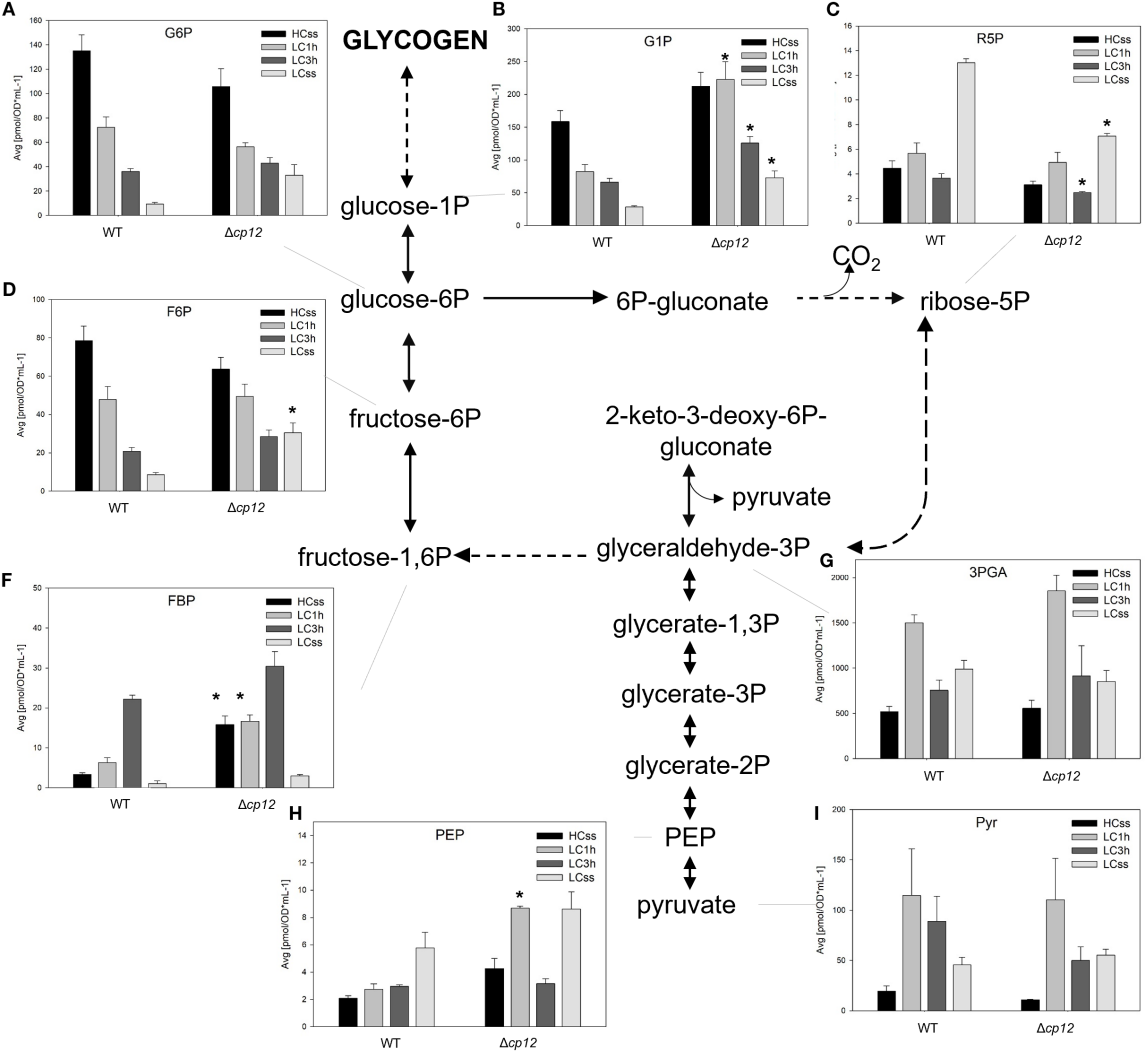
Schematic presentation of the glycolysis and oxidative pentose-phosphate (OPP) pathway and graphs showing the concentration of intermediates under different Ci conditions. Cells of the *Synechocystis* sp. PCC 6803 wild type (WT) and the mutant Δ*cp12* were grown at either 5% CO_2_ (HCss) or ambient 0.04% CO_2_ (LCss), and cells acclimated to HC were shifted to LC conditions (LC1h or LC3h). CBB cycle intermediates were quantified using GC-MS and the values [panels **(A–I)**] are displayed as pmol OD750–^1^ ml^-1^ (mean values from two independent experiments with each four biological replicates and standard deviations, statistically significant differences (p ≤ 0.05) between WT and mutant are marked with asterisks). Abbreviations and further details are explained in **Figure 1**.

Next, we analyzed changes in metabolites of sugar catabolism via the OPP pathway and classical glycolysis in the two strains under different Ci conditions. Many metabolites of these pathways, such as F6P, FBP, R5P and 3PGA (**Figures 4C–F, G**), are shared with the CBB cycle; hence, their changes discussed in the previous section may also account for differences in the glycogen catabolism in the two strains under different Ci conditions.

The OPP pathway is known as the main route for glucose catabolism under dark but also light conditions (reviewed in Lucius and Hagemann, 2024). The amount of glucose 6-phosphate (G6P), the compound at the beginning of this pathway, showed almost similar stepwise declines when cells of the two strains were shifted from HC to LC conditions (**Figure 4A**). However, the steady-state amount of G6P was clearly higher in LC-grown cells of the mutant Δ*cp12*, as was also observed for glucose 1-phosphate (G1P). Interestingly, there was no decrease of G1P when mutant cells were shifted from HC to LC conditions for 1 h (**Figure 4B**). This delayed response of G1P in mutant compared to WT cells is consistent with the delayed glycogen consumption after LC shifts (**Figure 3**) and lower activity of G6PDH (Lucius et al., 2022) in Δ*cp12*.

**Figure 5:**
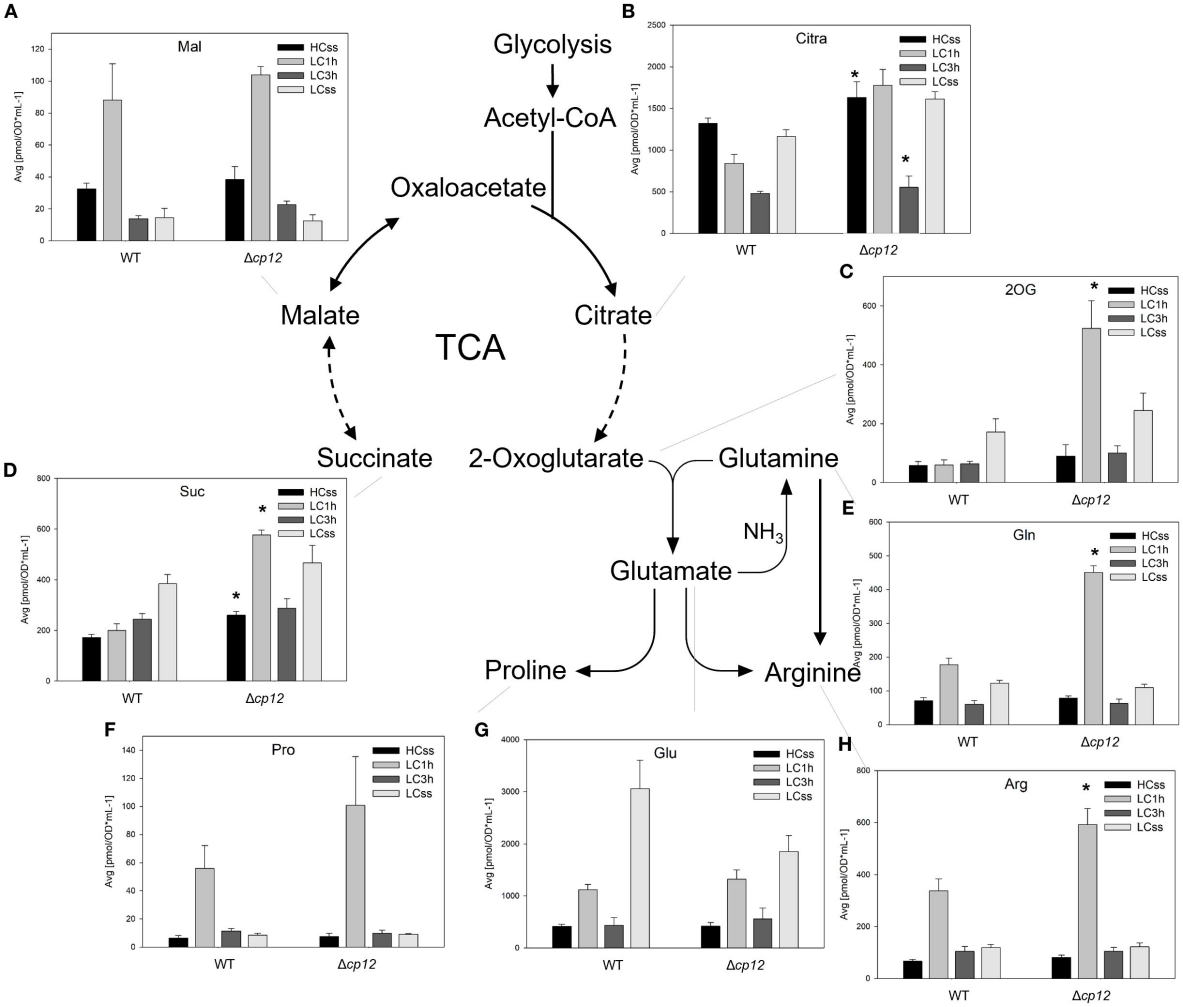
Schematic presentation of the open tricarboxylic acid (TCA) cycle and associated reactions and graphs showing the concentration of intermediates under different Ci conditions. Cells of the *Synechocystis* sp. PCC 6803 wild type (WT) and the mutant Δ*cp12* were grown at either 5% CO_2_ (HCss) or ambient 0.04% CO_2_ (LCss), and cells acclimated to HC were shifted to LC conditions (LC1h or LC3h). CBB cycle intermediates were quantified using GC-MS and the values [panels **(A–H)**] are displayed as pmol OD750–^1^ ml^-1^ (mean values from two independent experiments with each four biological replicates and standard deviations, statistically significant differences (p ≤ 0.05) between WT and mutant are marked with asterisks). Abbreviations and further details are explained in **Figure 1**.

Furthermore, significant alterations in the metabolites of lower glycolysis were observed in the two strains under varying Ci conditions. The levels of phosphoenolpyruvate (PEP) and pyruvate were found to be elevated in both strains under steady-state LC conditions (**Figures 4H, I**), which is consistent with an enhanced export of organic carbon from the CBB cycle towards lower glycolysis under Ci-limiting conditions (e.g., Eisenhut et al., 2008). In these conditions, carbon is less utilized for glycogen synthesis but predominantly for sustaining nitrogen assimilation. Interestingly, a transient accumulation of PEP occurred 1 h after LC shift in cells of the mutant Δ*cp12* that might reflect a higher export of organic carbon to lower glycolysis and the open TCA cycle (see **Figure 5**) in this strain at this time point. The levels of 3PGA showed similar alterations in both strains (**Figure 4G**).

### TCA cycle and associated intermediates of nitrogen metabolism

In cyanobacteria, the TCA cycle is not closed due to the absence of the 2-oxoglutarate-(2OG)-dehydrogenase complex. Therefore, the oxidative branch is mainly utilized to synthesize 2OG as a carbon acceptor for nitrogen assimilation in the glutamine synthetase/glutamine oxoglutarate aminotransferase/glutamate synthase (GS/GOGAT) cycle. In contrast, the reductive branch produces succinate that can serve as substrate in respiration. Two shunts have been described that can close the cyanobacterial TCA cycle converting 2OG into succinate either using the enzymes 2-oxoglutarate decarboxylase and succinic semialdehyde dehydrogenase (Zhang and Bryant, 2011) or the γ-aminobutyrate aminotransferase (Xiong et al., 2014); however, the flux through these shunts seemed to be low under steady-state conditions.

Metabolites of the oxidative branch such as citrate/isocitrate (Cit) showed no strong deviations under HC and LC steady-state conditions in the two strains with the exception of 2OG, which non-significantly increased under LC steady-state conditions in both strains (**Figures 5B, C**). Moreover, we observed a strong peak of 2OG amounts 1 h after LC shift in mutant Δ*cp12*. This high accumulation was also observed for glutamine (**Figure 5E**), the ammonia assimilation product of the glutamine synthetase reaction using 2OG as an acceptor molecule. Increased levels were also observed for arginine and proline at this time point in mutant cells (**Figures 5F, H**), which may result from the more available glutamine pool in transaminase reactions. In contrast, the amount of glutamate increased in both strains under LC compared to HC, reaching higher levels in the WT (**Figure 5G**).

Malate from the reductive branch showed basically the same alterations in the two strains, a substantial transient accumulation after 1 h LC shift (**Figure 5A**), as was observed for pyruvate in the lower glycolysis (see **Figure 4I**). Then, malate decreases to lower levels in LC-acclimated cells of both strains. Succinate was rather increasing with higher trends in the mutant Δ*cp12* (**Figure 5D**).

## Discussion

As expected, the Ci availability had a marked impact on the primary carbon metabolism in *Synechocystis*. In the presence of high Ci, the oxygenation reaction of RubisCO is much lower, as depicted in the low steady-state levels of the photorespiratory intermediate 2PG, than under LC (see **Figure 2**), despite the induction of the CCM after the shift from HC to LC conditions. The fast and strong response of 2PG levels to alternating Ci levels makes this intermediate an excellent candidate to monitor the carbon availability of cyanobacterial cells, which influences the activity of the CCM-regulating transcription factors NdhR (Jiang et al., 2018) and CmpR (Nishimura et al., 2008). Moreover, the absence of CP12 had marked impact on carbon partitioning. Under HC conditions gluconeogenesis was stimulated resulting in elevated glycogen storage that is consumed after a shift to LC to replenish the central carbon metabolism. In addition to the CBB, intermediates of other metabolic pathways exhibit alterations in their steady-state values, reflecting different carbon fluxes under fluctuating Ci conditions (e.g., Eisenhut et al., 2008; Huege et al., 2011). The increased glycogen pool in cells of mutant Δ*cp12* is likely a consequence of an enhanced activity of the regenerative phase of the CBB cycle. Our previous study showed that even in the light part of GapDH2 is associated with CP12 (Lucius et al., 2022); hence, the absence of the inhibitory CP12 interaction resulted in full activity of GapDH2 and PRK to regenerate RuBP under HC. Accordingly, the turnover of glycogen is delayed, most likely because less organic carbon from the glycogen pool is used to replenish the CBB cycle compared to WT cells.

Most of the principal metabolic changes found in WT cells exposed to different Ci conditions remained visible in the cells of the mutant Δ*cp12*; however, in many cases, the alterations were more pronounced in mutant cells, particularly in the transient situation, i.e., 1 h after shift from HC to LC conditions, when the acclimation process just started. Such a behavior was expected in a mutant, in which a regulatory protein adjusting critical enzyme activities according to redox alterations is absent. Moreover, the widespread metabolic alterations in the mutant Δ*cp12* support the view that growth at different Ci conditions has a marked influence on the redox conditions in cyanobacterial cells, which is sensed by different redox regulating circuits, including the redox-dependent regulatory protein CP12. Similar conclusions were drawn in a recent study, in which the acclimation of *Synechocystis* to very high CO_2_ conditions of 30% (v/v) was analyzed (Carrasquer-Alvarez et al., 2025). The authors demonstrated that extremely high CO_2_ levels had a negative impact on the growth of *Synechocystis*, which was exacerbated in cells with reduced *cp12* expression due to metabolic pool imbalances. A previous study using a Δ*cp12* of *Synechococcus elongatus* provided evidence that the absence of CP12 had negative impact when grown at high light conditions, which was related to higher accumulation of harmful reactive oxygen species (ROS) in cells of the mutant compared to WT (Tamoi and Shigeoka, 2021).

As expected, we found alterations in the CBB cycle intermediates DHAP as proxy for Gap and RuBP (see **Figure 2**), which are the products of CP12 interaction partners GapDH2 and PRK. RuBP showed higher amounts in the mutant than WT cells when shifted for 1 h into LC, corresponding to the less inhibited PRK activity in the absence of CP12, whereas DHAP was significantly elevated only under LC steady-state conditions (see **Figure 2**). The overall metabolite pool changes suggest that the carbon metabolite regeneration portion is stalled under LC, likely due to low utilization of RuBP, which is consistent with the CBB limitation by Rubisco under low Ci conditions. This would lead to higher accumulation of metabolites both the fixation and reduction sides, including RuBP, DHAP, 3PGA.

Interestingly, the metabolites connected by the activity of the bifunctional fructose-1,6-bisphosphate/sedoheptulose-1,7-bisphosphate phosphatase (F/SBPase), FBP or SBP and F6P or S7P, respectively, showed an opposite behavior between mutant and WT under steady-state conditions. The amounts of the monophosphates F6P and S7P were lower in mutant than in WT cells under HC steady-state conditions, while those of the bisphosphates FBP and SBP were significantly elevated in the mutant at HC steady-state conditions. The conversion of FBP and SBP into F6P and S7P is catalyzed by a bifunctional F/SBPase in *Synechocystis* (García-Cañas et al., 2022; Caliebe et al., 2025), which might represent the rate limiting step in the regenerative phase of the CBB cycle (Miyagawa et al., 2001). The opposite relative changes of these metabolites is consistent with this view and indicates that the activity of the bifunctional F/SBPase might be directly or indirectly regulated by CP12 in *Synechocystis* under different Ci conditions.

In addition to the CBB cycle, intermediates of the glycolysis, OPP pathway, TCA cycle, and associated amino acids showed marked changes at different Ci, which were often different between the WT and mutant Δ*cp12*. In the OPP pathway, especially the amount of G1P was clearly elevated in the mutant compared to WT cells. In light of the slower glycogen consumption in mutant compared to WT cells (see **Figure 3**), this increase indicates rather a slower G1P consumption than its elevated synthesis from glycogen breakdown. However, we cannot rule out that the conversion of G1P into G6P by phosphoglucomutase might be also affected in the strain without CP12. Mixotrophic growth in the presence of glucose stimulated glycogen accumulation in WT and mutant Δ*cp12*, whereas the glycogen degradation was much slower in mutant cells when glucose was consumed (Lucius et al., 2022). Moreover, previous studies showed that the mutant Δ*cp12* is sensitive to external glucose when grown under light/dark cycles (Blanc-Garin et al., 2022; Lucius et al., 2022). The importance of CP12 and the associated GapDH2 and PRK enzymes for the utilization of glucose has also been observed in a recent CRISPRi screen, in which repression of these three proteins had marked negative fitness scores on the growth of *Synechocystis* under mixotrophic conditions (Miao et al., 2023). Interestingly, the study of Miao et al. (2023) also showed a negative impact of GapDH2 and CP12 downregulation on fitness under low Ci conditions, whereas we did not detect slower growth of mutant Δ*cp12* compared to WT when grown at either HC or LC conditions (Lucius et al., 2022). Global changes in the overall metabolism as summarized in **Figure 1**, including alterations in the metabolites of the primary carbon and nitrogen metabolism, have also been reported for tobacco plants with strong antisense-mediated repression of the chloroplastidial CP12 amounts (Howard et al., 2011).

Collectively, our results indicate that the redox-controlled CP12 protein has a more widespread impact on many intermediates of both primary carbon and nitrogen metabolism. The question arises whether these changes are directly or indirectly linked to the absence of CP12. Previously, we demonstrated that the glucose sensitivity of the mutant Δ*cp12* is primarily due to the absence of redox-dependent regulation of GapDH2 rather than PRK (Lucius et al., 2022). Recently, Nishiguchi et al. (2025) analyzed *Synechocystis* strains expressing PRK variants with exchanged cysteine residues in its C-terminal part that are no longer directly redox-regulated. The metabolic analysis of these mutants revealed an elevated accumulation of organic acids within the TCA cycle, including malate, fumarate, succinate, and citrate. We also found higher succinate and citrate levels in mutant Δ*cp12* compared to WT 1 h after shift from HC to LC, which, according to the results of Nishiguchi et al. (2025) might be related to the deregulation of PRK rather than GapDH2. In another study, the role of the glucosyltransferase Slr1064 for cell wall and carbon metabolism, especially effects linked to UDP-N-acetylglucosamine, was analyzed in *Synechocystis* (Han et al., 2024). The authors reported changes in CP12 contents and growth repression of the mutant Δ*slr1064* in the presence of glucose, which could be compensated by the overexpression of GapDH2 (Han et al., 2024). Hence, glucose metabolism linked to glycogen and cell wall synthesis might be rather dependent on the CP12-mediated regulation of GapDH2 activity. However, we cannot rule out that CP12 has other targets or moonlighting functions. In *Chlamydomonas*, CP12 can also bind aldolase (Erales et al., 2008) or can act in a chaperone-like manner to stabilize enzymes (Erales et al., 2009). Finally, Gérard et al. (2022) demonstrated that the proteome of the *Chlamydomonas cp12* mutant exhibited numerous differences, including significant changes in enzymes involved in primary carbon and nitrogen metabolism. Such changes will undoubtedly also have a significant impact on the regulation of primary metabolism.

## Data Availability

The original contributions presented in the study are included in the article/**Supplementary Material**. Further inquiries can be directed to the corresponding author.
