# Supplementary material for: The impact of CP12 on the metabolome of cyanobacteria under fluctuating CO2 conditions
Source: Front Plant Sci. 2025 Sep 23;16:1674721. doi: 10.3389/fpls.2025.1674721 (PMC12500658; doi:10.3389/fpls.2025.1674721)
Supplement: Supplementary file 1 [file DataSheet1.pdf]

Supplementary data for the manuscript

The impact of CP12 on the metabolome of cyanobacteria under fluctuating CO_2 conditions

Stefan Lucius[1]*, Stéphanie Arrivault[2]*, Regina Feil[2], Luna Alvarenga-Lucius[1], Martin Hagemann[1,3]

[1]Plant Physiology, Institute of Biosciences, University of Rostock, Albert-Einstein-Str. 3, 18059 Rostock, Germany

[2]Max Planck Institute of Molecular Plant Physiology, Am Mühlenberg 1, 14476 Potsdam-Golm, Germany

[3]Interdisciplinary Faculty, Department Life, Light and Matter, University of Rostock, Albert-Einstein-Str. 28, 18059 Rostock, Germany

*equally contributing authors

corresponding author: Martin Hagemann; University of Rostock, Institute of Biosciences, Dept. Plant Physiology, Albert-Einstein-Str. 3, D-18059 Rostock, Germany, Tel. +49(0)3814986110, Fax. +49(0)3814986112, Email: martin.hagemann@uni-rostock.de

Figure

Figure S1- Principal component analysis (PCA) with self-organizing map (SOM) of metabolite data

Tables

Table S1 – Mean values of metabolome data per cell density

Table S2 – Calculated mean values of cellular metabolite concentrations

A:

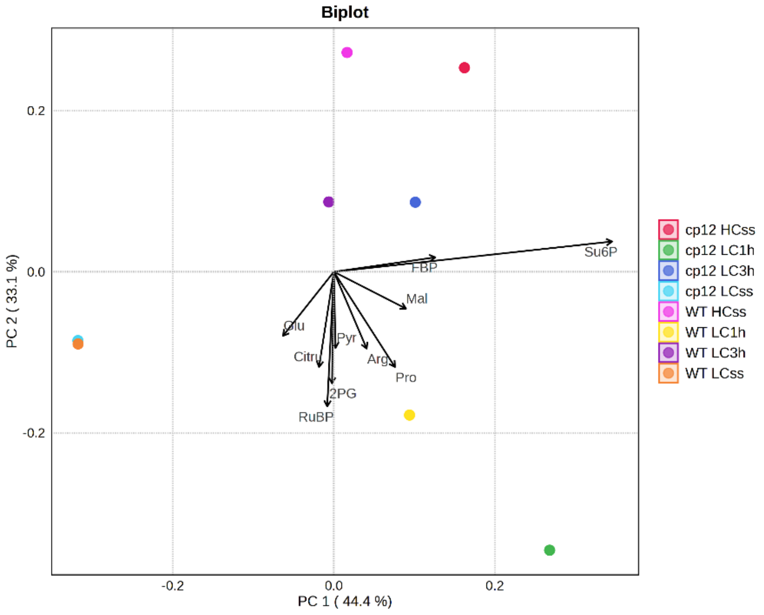
Biplot
0.2
0.0
-0.2
PC 2 (33.1 %)
-0.2
0.0
0.2
PC 1 (44.4 %)
Su6P
FBP
Mal
Glu
Pyr
Citr
Arg
Pro
2PG
RuBP
cp12 HCss
cp12 LC1h
cp12 LC3h
cp12 LCss
WT HCss
WT LC1h
WT LC3h
WT LCss

B:

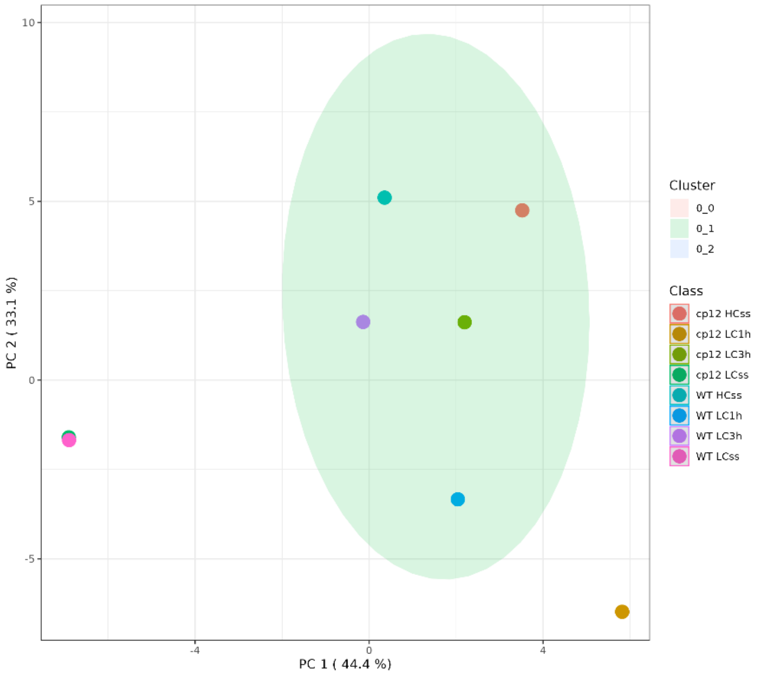
10
5
0
-5
PC 2 (33.1 %)
-4
0
4
PC 1 (44.4 %)
Cluster
0_0
0_1
0_2
Class
cp12 HCss
cp12 LC1h
cp12 LC3h
cp12 LCss
WT HCss
WT LC1h
WT LC3h
WT LCss

C:

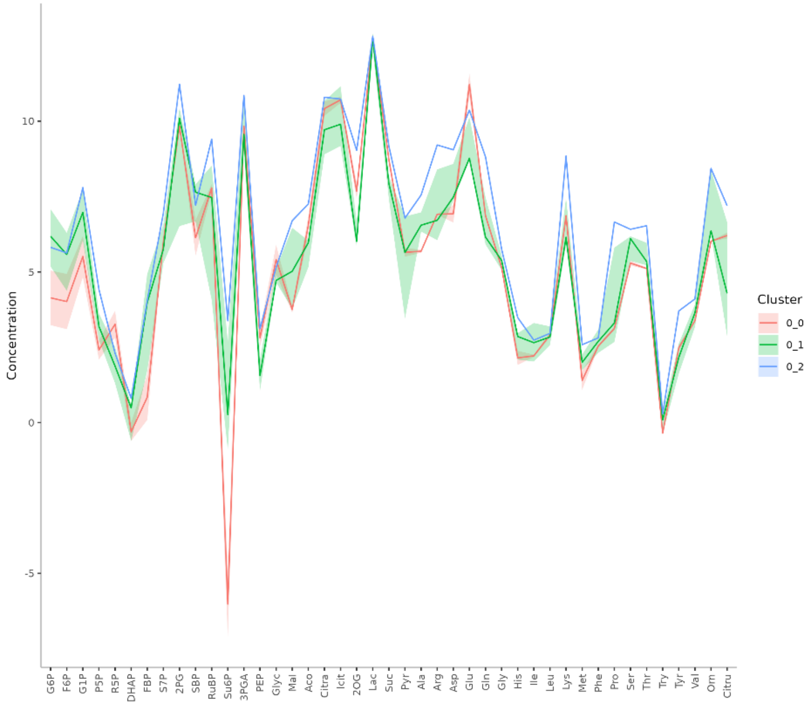

Figure S1: Principal component analysis (PCA) with self-organizing map (SOM) clustering for metabolites in response to different carbon concentrations. A: PCA shows 10 principal metabolites that explain the higher percentage of variance in the dataset. **B**: SOM space shows two principal components (PC1 and PC2) that explain the higher percentage of variance in the dataset, and represent the clustering patterns of the metabolic profile in response to carbon (indicated by different colors). **C**: Variance among all metabolites in the different clusters in response to carbon.

Table S1: Quantified metabolites in cells of the wild type (WT) and the mutant Δ*cp12* grown at different inorganic carbon conditions.

Shown are mean values in pmol per ml cell suspension and its optical density measured at 750 nm (OD) and standard errors (StdErr) (n = 6). Statistics were performed using Student's t-test. Statistical significant p values are marked in yellow. (HC – high CO_2 of 5% in air (v/v), LC- low CO_2 of ambient air, 0.04%)

**Phosphorylated intermediates**	**HC steady state**		**LC 1h**		**LC 3h**		**LC steady state**	
**glucose 6-phosphate**	[pmol/OD*mL]	StdErr	[pmol/OD*mL]	StdErr	[pmol/OD*mL]	StdErr	[pmol/OD*mL]	StdErr
WT	135.134	13.023	72.525	8.383	36.192	2.283	9.416	1.387
cp12	105.797	14.505	56.307	3.547	42.855	4.603	32.998	8.653
**t-statistic**	1.505		1.782		-1.297		-2.691	
**degrees of freedom**		9.886		6.734		7.319		5.257
**P value**	**0.333**		**0.250**		**0.472**		**0.086**	
**fructose 6-phosphate**	[pmol/OD*mL]	StdErr	[pmol/OD*mL]	StdErr	[pmol/OD*mL]	StdErr	[pmol/OD*mL]	StdErr
WT	78.519	7.562	47.853	6.714	20.836	1.909	8.592	1.124
cp12	63.668	6.087	49.497	6.414	28.548	3.392	30.499	5.113
**t-statistic**	1.530		-0.177		-1.981		-4.185	
**degrees of freedom**		9.564		9.979		7.879		5.482
**P value**	**0.321**		**1.727**		**0.176**		**0.017**	
**glucose 1-phosphate**	[pmol/OD*mL]	StdErr	[pmol/OD*mL]	StdErr	[pmol/OD*mL]	StdErr	[pmol/OD*mL]	StdErr
WT	158.587	16.854	82.526	10.897	66.423	5.309	28.521	1.832
cp12	212.647	20.888	222.761	27.007	125.833	9.941	73.022	10.620
**t-statistic**	-2.014		-4.815		-5.272		-4.129	
**degrees of freedom**		9.572		6.586		7.638		5.297
**P value**	**0.150**		**0.006**		**0.002**		**0.018**	
**ribulose 5-P + xylulose 5-P**	[pmol/OD*mL]	StdErr	[pmol/OD*mL]	StdErr	[pmol/OD*mL]	StdErr	[pmol/OD*mL]	StdErr
WT	6.692	0.795	12.332	2.754	7.210	1.249	4.282	0.618
cp12	9.371	0.894	21.384	2.074	9.127	0.723	6.623	0.781
**t-statistic**	-2.239		-2.626		-1.329		-2.349	
**degrees of freedom**		9.865		9.292		8.011		9.497

**P value**	**0.104**		**0.055**		**0.441**		**0.087**	
**ribose 5-phosphate**	[pmol/OD*mL]	StdErr	[pmol/OD*mL]	StdErr	[pmol/OD*mL]	StdErr	[pmol/OD*mL]	StdErr
WT	4.457	0.612	5.665	0.855	3.670	0.347	13.027	0.315
cp12	3.117	0.286	4.936	0.798	2.483	0.083	7.069	0.228
**t-statistic**	1.984		0.623		3.330		15.339	
**degrees of freedom**		7.085		9.954		5.578		9.110
**P value**	**0.175**		**1.098**		**0.042**		**0.000**	
**dihydroxyacetonephosphate**	[pmol/OD*mL]	StdErr	[pmol/OD*mL]	StdErr	[pmol/OD*mL]	StdErr	[pmol/OD*mL]	StdErr
WT	0.657	0.148	1.452	0.376	1.403	0.187	0.658	0.040
cp12	0.738	0.148	1.750	0.282	1.490	0.182	1.000	0.065
**t-statistic**	-0.386		-0.634		-0.332		-4.468	
**degrees of freedom**		10.000		9.272		9.994		8.345
**P value**	**1.417**		**1.084**		**1.494**		**0.004**	
**fructose 1.6-bisphosphate**	[pmol/OD*mL]	StdErr	[pmol/OD*mL]	StdErr	[pmol/OD*mL]	StdErr	[pmol/OD*mL]	StdErr
WT	3.330	0.452	6.308	1.210	22.236	0.994	1.067	0.644
cp12	15.846	2.116	16.623	1.572	30.434	3.664	3.017	0.282
**t-statistic**	-5.785		-5.200		-2.159		-2.776	
**degrees of freedom**		5.456		9.384		5.732		6.853
**P value**	**0.004**		**0.001**		**0.166**		**0.064**	
sedoheptulose 7-phosphate	[pmol/OD*mL]	StdErr	[pmol/OD*mL]	StdErr	[pmol/OD*mL]	StdErr	[pmol/OD*mL]	StdErr
WT	81.375	3.653	95.481	12.543	39.384	4.153	65.877	7.146
cp12	54.002	5.282	123.947	22.118	51.872	7.119	62.624	12.037
**t-statistic**	4.262		-1.119		-1.515		0.232	
**degrees of freedom**		8.893		7.914		8.050		8.135
**P value**	**0.006**		**0.600**		**0.336**		**1.644**	
**2-phosphoglycolate**	[pmol/OD*mL]	StdErr	[pmol/OD*mL]	StdErr	[pmol/OD*mL]	StdErr	[pmol/OD*mL]	StdErr
WT	91.875	6.009	1383.628	146.549	1093.620	109.494	925.792	131.765
cp12	129.562	13.029	2397.911	207.557	1290.560	221.614	904.785	221.279
**t-statistic**	-2.627		-3.992		-0.797		0.082	
**degrees of freedom**		7.035		8.993		7.304		8.150
**P value**	**0.068**		**0.008**		**0.904**		**1.874**	

**sedoheptulose1.7-bisphosphate**	[pmol/OD*mL]	StdErr	[pmol/OD*mL]	StdErr	[pmol/OD*mL]	StdErr	[pmol/OD*mL]	StdErr
WT	102.418	26.095	109.493	5.450	200.117	25.735	46.807	8.259
cp12	232.093	26.300	148.480	12.952	239.958	30.404	105.154	11.164
**t-statistic**	-3.500		-2.774		-1.000		-4.202	
**degrees of freedom**		9.999		6.717		9.734		9.212
**P value**	**0.013**		**0.064**		**0.687**		**0.005**	
**ribulose 1.5-bisphosphate**	[pmol/OD*mL]	StdErr	[pmol/OD*mL]	StdErr	[pmol/OD*mL]	StdErr	[pmol/OD*mL]	StdErr
WT	16.838	5.323	367.468	86.979	198.516	25.516	203.743	41.775
cp12	22.268	3.258	679.390	96.040	177.132	21.588	240.154	38.120
**t-statistic**	-0.870		-2.407		0.640		-0.644	
**degrees of freedom**		8.285		9.903		9.733		9.917
**P value**	**0.819**		**0.079**		**1.076**		**1.071**	
**sucrose 6-phosphate**	[pmol/OD*mL]	StdErr	[pmol/OD*mL]	StdErr	[pmol/OD*mL]	StdErr	[pmol/OD*mL]	StdErr
WT	1.078	0.073	1.199	0.127	0.538	0.029	0.033	0.034
cp12	6.317	0.531	10.414	0.314	2.633	0.364	0.007	0.029
**t-statistic**	-9.766		-27.233		-5.741		0.573	
**degrees of freedom**		5.188		6.598		5.066		9.784
**P value**	**0.000**		**0.000**		**0.004**		**1.162**	
**3-Phosphoglycerate**	[pmol/OD*mL]	StdErr	[pmol/OD*mL]	StdErr	[pmol/OD*mL]	StdErr	[pmol/OD*mL]	StdErr
WT	520.283	55.737	1501.339	88.319	755.336	112.787	987.700	99.580
cp12	555.352	89.609	1854.567	173.495	914.680	333.152	852.268	122.349
**t-statistic**	-0.332		-1.814		-0.453		0.859	
**degrees of freedom**		8.365		7.428		6.131		9.604
**P value**	**1.496**		**0.225**		**1.333**		**0.826**	
**PEP phosphoenolpyruvate**	[pmol/OD*mL]	StdErr	[pmol/OD*mL]	StdErr	[pmol/OD*mL]	StdErr	[pmol/OD*mL]	StdErr
WTC	2.094	0.179	2.727	0.402	2.955	0.116	5.763	1.165
cp12	4.262	0.748	8.681	0.140	3.169	0.353	8.610	1.276
**t-statistic**	-2.820		-13.986		-0.576		-1.648	
**degrees of freedom**		5.574		6.188		6.068		9.917
**P value**	**0.074**		**0.000**		**1.171**		**0.267**	

Organic acids	HC steady state		LC 1h		LC 3h		LC steady state	
**glycerate**	[pmol/OD*mL]	StdErr	[pmol/OD*mL]	StdErr	[pmol/OD*mL]	StdErr	[pmol/OD*mL]	StdErr
WT	29.402	2.816	34.460	4.015	25.860	3.746	59.386	2.533
cp12	25.192	4.365	35.641	3.283	26.309	3.274	30.405	3.676
**t-statistic**	0.810		-0.228		-0.090		6.492	
**degrees of freedom**		8.548		9.621		9.824		8.875
**P value**	**0.882**		**1.650**		**1.860**		**0.000**	
**malate**	[pmol/OD*mL]	StdErr	[pmol/OD*mL]	StdErr	[pmol/OD*mL]	StdErr	[pmol/OD*mL]	StdErr
WT	32.466	3.763	88.139	22.794	13.749	1.954	14.541	5.734
cp12	38.331	8.193	103.892	5.150	22.574	2.290	12.499	3.727
**t-statistic**	-0.651		-0.674		-2.931		0.299	
**degrees of freedom**		7.020		5.509		9.758		8.584
**P value**	**1.072**		**1.060**		**0.033**		**1.546**	
**Aconitic Acid**	[pmol/OD*mL]	StdErr	[pmol/OD*mL]	StdErr	[pmol/OD*mL]	StdErr	[pmol/OD*mL]	StdErr
WT	65.374	7.251	62.696	15.234	35.708	1.694	76.391	14.335
cp12	65.613	11.545	152.638	24.103	39.480	9.877	114.941	19.450
**t-statistic**	-0.018		-3.154		-0.376		-1.595	
**degrees of freedom**		8.413		8.445		5.294		9.194
**P value**	**1.973**		**0.027**		**1.444**		**0.290**	
**Citric Acid**	[pmol/OD*mL]	StdErr	[pmol/OD*mL]	StdErr	[pmol/OD*mL]	StdErr	[pmol/OD*mL]	StdErr
WT	1319.362	63.420	840.824	106.851	478.876	25.108	1162.450	82.271
cp12	1633.210	186.369	1777.476	192.381	554.125	133.884	1612.496	87.279
**t-statistic**	-1.594		-4.256		-0.552		-3.752	
**degrees of freedom**		6.143		7.817		5.351		9.965
**P value**	**0.324**		**0.008**		**1.209**		**0.009**	
**Isocitric Acid**	[pmol/OD*mL]	StdErr	[pmol/OD*mL]	StdErr	[pmol/OD*mL]	StdErr	[pmol/OD*mL]	StdErr
WT	2285.777	91.126	954.723	134.137	579.465	76.782	1559.170	80.818
cp12	2011.877	193.518	1706.038	150.465	633.149	64.048	1795.550	89.475
**t-statistic**	1.281		-3.727		-0.537		-1.961	
**degrees of freedom**		7.113		9.871		9.688		9.898
**P value**	**0.482**		**0.009**		**1.209**		**0.163**	
**2-Ketoglutaric Acid**	[pmol/OD*mL]	StdErr	[pmol/OD*mL]	StdErr	[pmol/OD*mL]	StdErr	[pmol/OD*mL]	StdErr

WT	57.861	13.387	59.684	16.987	64.598	8.048	171.711	45.308
cp12	89.468	38.986	524.111	93.902	100.061	24.577	244.799	59.378
**t-statistic**	-0.767		-4.867		-1.371		-0.979	
**degrees of freedom**		6.163		5.327		6.060		9.348
**P value**	**0.945**		**0.009**		**0.439**		**0.707**	
**Lactic Acid**	[pmol/OD*mL]	StdErr	[pmol/OD*mL]	StdErr	[pmol/OD*mL]	StdErr	[pmol/OD*mL]	StdErr
WT	7677.088	249.021	5337.094	312.604	6837.352	406.543	7449.174	226.032
cp12	6454.309	582.400	7233.468	284.494	7044.251	654.975	6157.390	669.520
**t-statistic**	1.930		-4.487		-0.268		1.828	
**degrees of freedom**		6.769		9.913		8.355		6.125
**P value**	**0.204**		**0.003**		**1.590**		**0.235**	
**succinate**	[pmol/OD*mL]	StdErr	[pmol/OD*mL]	StdErr	[pmol/OD*mL]	StdErr	[pmol/OD*mL]	StdErr
WT	172.389	11.454	199.722	26.622	244.212	21.681	384.204	37.083
cp12	260.508	15.341	576.313	19.478	287.902	38.112	466.772	68.643
**t-statistic**	-4.603		-11.416		-0.996		-1.058	
**degrees of freedom**		9.253		9.161		7.929		7.689
**P value**	**0.003**		**0.000**		**0.704**		**0.650**	
**pyruvate**	[pmol/OD*mL]	StdErr	[pmol/OD*mL]	StdErr	[pmol/OD*mL]	StdErr	[pmol/OD*mL]	StdErr
WT	19.482	5.318	114.539	46.292	88.831	24.949	45.532	7.419
cp12	10.990	0.470	110.427	40.994	49.923	13.561	55.396	5.785
**t-statistic**	1.591		0.066		1.370		-1.048	
**degrees of freedom**		5.078		9.856		7.717		9.439
**P value**	**0.345**		**1.897**		**0.426**		**0.644**	
**Amino acids**	**HC steady state**		**LC 1h**		**LC 3h**		**LC steady state**	
**Alanine**	[pmol/OD*mL]	StdErr	[pmol/OD*mL]	StdErr	[pmol/OD*mL]	StdErr	[pmol/OD*mL]	StdErr
WT	81.074	9.387	125.771	57.116	86.465	10.285	53.166	5.221
cp12	93.669	11.300	187.486	31.481	97.715	16.518	49.824	3.232
**t-statistic**	-0.857		-0.946		-0.578		0.544	
**degrees of freedom**		9.675		7.781		8.370		8.342
**P value**	**0.827**		**0.751**		**1.158**		**1.202**	
**Arginine**	[pmol/OD*mL]	StdErr	[pmol/OD*mL]	StdErr	[pmol/OD*mL]	StdErr	[pmol/OD*mL]	StdErr

WT	66.526	6.457	337.441	45.634	104.800	18.702	119.104	12.176
cp12	80.874	9.559	592.893	60.841	105.136	14.662	121.947	15.603
**t-statistic**	-1.244		-3.359		-0.014		-0.144	
**degrees of freedom**		8.776		9.273		9.461		9.442
**P value**	**0.498**		**0.017**		**1.978**		**1.778**	
**Aspartic Acid**	[pmol/OD*mL]	StdErr	[pmol/OD*mL]	StdErr	[pmol/OD*mL]	StdErr	[pmol/OD*mL]	StdErr
WT	174.553	7.311	383.637	26.993	178.520	27.922	99.918	17.041
cp12	164.647	17.973	530.532	61.085	187.057	39.324	148.388	17.596
**t-statistic**	0.511		-2.200		-0.177		-1.979	
**degrees of freedom**		6.610		6.881		9.020		9.990
**P value**	**1.256**		**0.140**		**1.727**		**0.158**	
**Glutamic Acid**	[pmol/OD*mL]	StdErr	[pmol/OD*mL]	StdErr	[pmol/OD*mL]	StdErr	[pmol/OD*mL]	StdErr
WT	415.157	39.444	1118.541	103.470	437.025	146.306	3058.236	549.034
cp12	418.690	68.827	1320.302	177.234	556.513	211.936	1850.100	309.723
**t-statistic**	-0.045		-0.983		-0.464		1.917	
**degrees of freedom**		7.965		8.054		8.884		7.890
**P value**	**1.931**		**0.709**		**1.310**		**0.194**	
**Glutamine**	[pmol/OD*mL]	StdErr	[pmol/OD*mL]	StdErr	[pmol/OD*mL]	StdErr	[pmol/OD*mL]	StdErr
WT	71.273	8.965	177.297	20.073	60.404	11.421	122.911	8.282
cp12	78.595	7.058	450.668	19.508	63.076	13.364	110.125	9.985
**t-statistic**	-0.642		-9.767		-0.152		0.986	
**degrees of freedom**		9.478		9.992		9.763		9.669
**P value**	**1.074**		**0.000**		**1.765**		**0.700**	
**Glycine**	[pmol/OD*mL]	StdErr	[pmol/OD*mL]	StdErr	[pmol/OD*mL]	StdErr	[pmol/OD*mL]	StdErr
WT	46.847	9.816	41.612	0.646	33.455	7.433	37.277	1.962
cp12	42.690	14.282	54.089	4.344	41.685	7.051	33.548	2.119
**t-statistic**	0.240		-2.841		-0.803		1.291	
**degrees of freedom**		8.862		5.221		9.972		9.941
**P value**	**1.633**		**0.072**		**0.885**		**0.457**	
**Histidine**	[pmol/OD*mL]	StdErr	[pmol/OD*mL]	StdErr	[pmol/OD*mL]	StdErr	[pmol/OD*mL]	StdErr
WT	4.257	1.878	7.807	0.329	7.251	2.886	5.204	0.924
cp12	7.390	5.186	11.187	0.850	6.098	1.922	3.774	0.509

**t-statistic**	-0.568		-3.708		0.333		1.355	
**degrees of freedom**		6.290		6.468		8.705		7.779
**P value**	**1.181**		**0.020**		**1.496**		**0.435**	
**Isoleucine**	[pmol/OD*mL]	StdErr	[pmol/OD*mL]	StdErr	[pmol/OD*mL]	StdErr	[pmol/OD*mL]	StdErr
WT	4.724	0.591	4.085	0.305	9.884	1.980	4.529	0.451
cp12	6.284	1.282	6.663	0.392	6.920	0.618	4.763	0.279
**t-statistic**	-1.105		-5.193		1.429		-0.441	
**degrees of freedom**		7.033		9.436		5.967		8.352
**P value**	**0.612**		**0.001**		**0.425**		**1.342**	
**Leucine**	[pmol/OD*mL]	StdErr	[pmol/OD*mL]	StdErr	[pmol/OD*mL]	StdErr	[pmol/OD*mL]	StdErr
WT	5.953	0.660	6.468	0.077	9.127	2.290	7.889	0.507
cp12	7.416	1.741	7.786	0.683	7.164	1.170	7.137	0.214
**t-statistic**	-0.786		-1.917		0.764		1.366	
**degrees of freedom**		6.406		5.126		7.445		6.731
**P value**	**0.924**		**0.227**		**0.940**		**0.442**	
**Lysine**	[pmol/OD*mL]	StdErr	[pmol/OD*mL]	StdErr	[pmol/OD*mL]	StdErr	[pmol/OD*mL]	StdErr
WT	70.970	10.319	174.908	19.743	60.092	9.219	123.286	8.246
cp12	78.356	7.573	459.748	26.204	63.762	11.674	110.546	10.167
**t-statistic**	-0.577		-8.682		-0.247		0.973	
**degrees of freedom**		9.175		9.293		9.490		9.591
**P value**	**1.156**		**0.000**		**1.621**		**0.712**	
**Methionine**	[pmol/OD*mL]	StdErr	[pmol/OD*mL]	StdErr	[pmol/OD*mL]	StdErr	[pmol/OD*mL]	StdErr
WT	3.995	0.601	4.206	0.124	3.340	0.449	2.127	0.179
cp12	4.788	0.667	6.004	0.460	3.395	0.128	3.267	0.298
**t-statistic**	-0.883		-3.773		-0.119		-3.283	
**degrees of freedom**		9.895		5.721		5.810		8.201
**P value**	**0.800**		**0.026**		**1.819**		**0.022**	
**Phenylalanine**	[pmol/OD*mL]	StdErr	[pmol/OD*mL]	StdErr	[pmol/OD*mL]	StdErr	[pmol/OD*mL]	StdErr
WT	5.695	0.339	4.980	0.053	8.475	2.007	5.997	0.504
cp12	8.449	1.013	7.018	0.638	6.485	0.356	5.638	0.320
**t-statistic**	-2.577		-3.184		0.976		0.601	
**degrees of freedom**		6.108		5.070		5.315		8.476

**P value**	**0.084**		**0.049**		**0.748**		**1.129**	
**Proline**	[pmol/OD*mL]	StdErr	[pmol/OD*mL]	StdErr	[pmol/OD*mL]	StdErr	[pmol/OD*mL]	StdErr
WT	6.400	1.814	55.941	16.276	11.445	1.844	8.401	1.291
cp12	7.473	2.506	100.814	34.786	9.888	2.210	9.045	0.564
**t-statistic**	-0.347		-1.168		0.541		-0.457	
**degrees of freedom**		9.111		7.089		9.688		6.843
**P value**	**1.473**		**0.562**		**1.203**		**1.327**	
**Serine**	[pmol/OD*mL]	StdErr	[pmol/OD*mL]	StdErr	[pmol/OD*mL]	StdErr	[pmol/OD*mL]	StdErr
WT	41.234	6.695	69.089	1.366	72.469	13.959	41.365	3.915
cp12	52.825	18.553	85.253	6.724	68.769	8.583	37.099	3.845
**t-statistic**	-0.588		-2.356		0.226		0.777	
**degrees of freedom**		6.280		5.412		8.308		9.997
**P value**	**1.156**		**0.130**		**1.654**		**0.914**	
**Threonine**	[pmol/OD*mL]	StdErr	[pmol/OD*mL]	StdErr	[pmol/OD*mL]	StdErr	[pmol/OD*mL]	StdErr
WT	40.739	4.013	62.410	0.981	37.288	4.408	33.929	3.047
cp12	42.418	9.977	92.545	3.107	40.668	6.880	35.308	4.210
**t-statistic**	-0.156		-9.250		-0.414		-0.265	
**degrees of freedom**		6.577		5.987		8.513		9.110
**P value**	**1.762**		**0.000**		**1.380**		**1.593**	
**Tryptophan**	[pmol/OD*mL]	StdErr	[pmol/OD*mL]	StdErr	[pmol/OD*mL]	StdErr	[pmol/OD*mL]	StdErr
WT	0.837	0.086	0.782	0.065	1.065	0.193	0.763	0.088
cp12	1.217	0.135	1.224	0.013	1.089	0.081	0.808	0.046
**t-statistic**	-2.368		-6.666		-0.117		-0.454	
**degrees of freedom**		8.465		5.398		6.709		7.566
**P value**	**0.091**		**0.002**		**1.822**		**1.328**	
**Tyrosine**	[pmol/OD*mL]	StdErr	[pmol/OD*mL]	StdErr	[pmol/OD*mL]	StdErr	[pmol/OD*mL]	StdErr
WT	3.098	0.277	4.481	0.253	4.534	1.102	5.953	1.044
cp12	6.140	1.353	13.039	0.371	4.320	0.309	5.250	0.660
**t-statistic**	-2.203		-19.044		0.186		0.570	
**degrees of freedom**		5.418		8.820		5.781		8.450
**P value**	**0.158**		**0.000**		**1.719**		**1.169**	
**Valine**	[pmol/OD*mL]	StdErr	[pmol/OD*mL]	StdErr	[pmol/OD*mL]	StdErr	[pmol/OD*mL]	StdErr

WT	9.018	1.153	14.225	0.761	15.179	3.938	10.259	0.944
cp12	10.671	3.037	17.188	2.769	12.479	2.412	10.554	0.414
**t-statistic**	-0.509		-1.032		0.585		-0.287	
**degrees of freedom**		6.411		5.750		8.288		6.856
**P value**	**1.258**		**0.699**		**1.150**		**1.568**	

**Others**	**HC steady state**		**LC 1h**		**LC 3h**		**LC steady state**	
**Ornithine**	[pmol/OD*mL]	StdErr	[pmol/OD*mL]	StdErr	[pmol/OD*mL]	StdErr	[pmol/OD*mL]	StdErr
WT	82.255	26.154	331.489	22.063	75.926	21.052	64.600	0.996
cp12	104.351	42.090	345.377	59.466	76.825	11.628	65.051	1.627
**t-statistic**	-0.446		-0.219		-0.037		-0.236	
**degrees of freedom**		8.360		6.351		7.791		8.287
**P value**	**1.335**		**1.668**		**1.942**		**1.638**	
**Citrulline**	[pmol/OD*mL]	StdErr	[pmol/OD*mL]	StdErr	[pmol/OD*mL]	StdErr	[pmol/OD*mL]	StdErr
WT	27.050	4.740	100.210	8.333	7.350	2.429	69.086	16.140
cp12	19.547	9.957	146.754	18.166	11.110	2.374	79.006	13.791
**t-statistic**	0.680		-2.329		-1.107		-0.467	
**degrees of freedom**		7.155		7.015		9.995		9.762
**P value**	**1.036**		**0.105**		**0.594**		**1.303**	

Table S2: Concentrations of quantified metabolites in cells of the wild type (WT) and the mutant ∆*cp12* grown at different inorganic carbon conditions.

Shown are mean values (Avg) of calculated cellular concentrations in mM and standard errors (StdErr) (n = 6). (HC – high CO_2 of 5% in air (v/v), LC- low CO_2 of ambient air, 0.04%)

**Phosphorylated intermediates**	**HC steady state**		**LC 1h**		**LC 3h**		**LC steady state**	
**glucose 6-phosphate**	Avg [mM]	StdErr	Avg [mM]	StdErr	Avg [mM]	StdErr	Avg [mM]	StdErr
WT	0.294	0.028	0.158	0.018	0.079	0.005	0.020	0.003
cp12	0.230	0.032	0.123	0.008	0.093	0.010	0.072	0.019
**fructose 6-phosphate**	Avg [mM]	StdErr	Avg [mM]	StdErr	Avg [mM]	StdErr	Avg [mM]	StdErr
WT	0.171	0.016	0.104	0.015	0.045	0.004	0.019	0.002
cp12	0.139	0.013	0.108	0.014	0.062	0.007	0.066	0.011
**glucose 1-phosphate**	Avg [mM]	StdErr	Avg [mM]	StdErr	Avg [mM]	StdErr	Avg [mM]	StdErr
WT	0.345	0.037	0.180	0.024	0.145	0.012	0.062	0.004
cp12	0.463	0.045	0.485	0.059	0.274	0.022	0.159	0.023
**ribulose 5-P + xylulose 5-P**	Avg [mM]	StdErr	Avg [mM]	StdErr	Avg [mM]	StdErr	Avg [mM]	StdErr
WT	0.015	0.002	0.027	0.006	0.016	0.003	0.009	0.001
cp12	0.020	0.002	0.047	0.005	0.020	0.002	0.014	0.002
**ribose 5-phosphate**	Avg [mM]	StdErr	Avg [mM]	StdErr	Avg [mM]	StdErr	Avg [mM]	StdErr
WT	0.010	0.001	0.012	0.002	0.008	0.001	0.028	0.001
cp12	0.007	0.001	0.011	0.002	0.005	0.000	0.015	0.000
**dihydroxyacetonephosphate**	Avg [mM]	StdErr	Avg [mM]	StdErr	Avg [mM]	StdErr	Avg [mM]	StdErr
WT	0.001	0.000	0.003	0.001	0.003	0.000	0.001	0.000
cp12	0.002	0.000	0.004	0.001	0.003	0.000	0.002	0.000
**fructose 1,6-bisphosphate**	Avg [mM]	StdErr	Avg [mM]	StdErr	Avg [mM]	StdErr	Avg [mM]	StdErr
WT	0.007	0.001	0.014	0.003	0.048	0.002	0.002	0.001

cp12	0.034	0.005	0.036	0.003	0.066	0.008	0.007	0.001
**sedoheptulose 7-phosphate**	Avg [mM]	StdErr	Avg [mM]	StdErr	Avg [mM]	StdErr	Avg [mM]	StdErr
WT	0.177	0.008	0.208	0.027	0.086	0.009	0.143	0.016
cp12	0.118	0.011	0.270	0.048	0.113	0.015	0.136	0.026
**2-phosphoglycolate**	Avg [mM]	StdErr	Avg [mM]	StdErr	Avg [mM]	StdErr	Avg [mM]	StdErr
WT	0.200	0.013	3.011	0.319	2.380	0.238	2.015	0.287
cp12	0.282	0.028	5.219	0.452	2.809	0.482	1.969	0.482
**sedoheptulose 1,7-bisphosphate**	Avg [mM]	StdErr	Avg [mM]	StdErr	Avg [mM]	StdErr	Avg [mM]	StdErr
WT	0.223	0.057	0.238	0.012	0.436	0.056	0.102	0.018
cp12	0.505	0.057	0.323	0.028	0.522	0.066	0.229	0.024
**ribulose 1,5-bisphosphate**	Avg [mM]	StdErr	Avg [mM]	StdErr	Avg [mM]	StdErr	Avg [mM]	StdErr
WT	0.037	0.012	0.800	0.189	0.432	0.056	0.443	0.091
cp12	0.048	0.007	1.479	0.209	0.385	0.047	0.523	0.083
**sucrose 6-phosphate**	Avg [mM]	StdErr	Avg [mM]	StdErr	Avg [mM]	StdErr	Avg [mM]	StdErr
WT	0.002	0.000	0.003	0.000	0.001	0.000	0.000	0.000
cp12	0.014	0.001	0.023	0.001	0.006	0.001	0.000	0.000
**3-Phosphoglycerate**	Avg [mM]	StdErr	Avg [mM]	StdErr	Avg [mM]	StdErr	Avg [mM]	StdErr
WT	1.132	0.121	3.267	0.192	1.644	0.245	2.150	0.217
cp12	1.209	0.195	4.036	0.378	1.991	0.725	1.855	0.266
**PEP phosphoenolpyruvate**	Avg [mM]	StdErr	Avg [mM]	StdErr	Avg [mM]	StdErr	Avg [mM]	StdErr
WTC	0.005	0.000	0.006	0.001	0.006	0.000	0.013	0.003
cp12	0.009	0.002	0.019	0.000	0.007	0.001	0.019	0.003

**Organic acids**	**HC steady state**		**LC 1h**		**LC 3h**		**LC steady state**	
**glycerate**	Avg [mM]	StdErr	Avg [mM]	StdErr	Avg [mM]	StdErr	Avg [mM]	StdErr

WT	0.064	0.006	0.075	0.009	0.056	0.008	0.129	0.006
cp12	0.055	0.010	0.078	0.007	0.057	0.007	0.066	0.008
**malate**	Avg [mM]	StdErr	Avg [mM]	StdErr	Avg [mM]	StdErr	Avg [mM]	StdErr
WT	0.071	0.008	0.192	0.050	0.030	0.004	0.032	0.012
cp12	0.083	0.018	0.226	0.011	0.049	0.005	0.027	0.008
**Aconitic Acid**	Avg [mM]	StdErr	Avg [mM]	StdErr	Avg [mM]	StdErr	Avg [mM]	StdErr
WT	0.142	0.016	0.136	0.033	0.078	0.004	0.166	0.031
cp12	0.143	0.025	0.332	0.052	0.086	0.021	0.250	0.042
**Citric Acid**	Avg [mM]	StdErr	Avg [mM]	StdErr	Avg [mM]	StdErr	Avg [mM]	StdErr
WT	2.871	0.138	1.830	0.233	1.042	0.055	2.530	0.179
cp12	3.554	0.406	3.868	0.419	1.206	0.291	3.509	0.190
**Isocitric Acid**	Avg [mM]	StdErr	Avg [mM]	StdErr	Avg [mM]	StdErr	Avg [mM]	StdErr
WT	4.974	0.198	2.078	0.292	1.261	0.167	3.393	0.176
cp12	4.378	0.421	3.713	0.327	1.378	0.139	3.908	0.195
**2-Ketoglutaric Acid**	Avg [mM]	StdErr	Avg [mM]	StdErr	Avg [mM]	StdErr	Avg [mM]	StdErr
WT	0.126	0.029	0.130	0.037	0.141	0.018	0.374	0.099
cp12	0.195	0.085	1.141	0.204	0.218	0.053	0.533	0.129
**Lactic Acid**	Avg [mM]	StdErr	Avg [mM]	StdErr	Avg [mM]	StdErr	Avg [mM]	StdErr
WT	16.707	0.542	11.615	0.680	14.880	0.885	16.211	0.492
cp12	14.046	1.267	15.742	0.619	15.330	1.425	13.400	1.457
**succinate**	Avg [mM]	StdErr	Avg [mM]	StdErr	Avg [mM]	StdErr	Avg [mM]	StdErr
WT	0.375	0.025	0.435	0.058	0.531	0.047	0.836	0.081
cp12	0.567	0.033	1.254	0.042	0.627	0.083	1.016	0.149
**pyruvate**	Avg [mM]	StdErr	Avg [mM]	StdErr	Avg [mM]	StdErr	Avg [mM]	StdErr
WT	0.042	0.012	0.249	0.101	0.193	0.054	0.099	0.016

cp12	0.024	0.001	0.240	0.089	0.109	0.030	0.121	0.013

**Amino acids**	**HC steady state**		**LC 1h**		**LC 3h**		**LC steady state**	
**Alanine**	Avg [mM]	StdErr	Avg [mM]	StdErr	Avg [mM]	StdErr	Avg [mM]	StdErr
WT	0.176	0.020	0.274	0.124	0.188	0.022	0.116	0.011
cp12	0.204	0.025	0.408	0.069	0.213	0.036	0.108	0.007
**Arginine**	Avg [mM]	StdErr	Avg [mM]	StdErr	Avg [mM]	StdErr	Avg [mM]	StdErr
WT	0.145	0.014	0.734	0.099	0.228	0.041	0.259	0.026
cp12	0.176	0.021	1.290	0.132	0.229	0.032	0.265	0.034
**Aspartic Acid**	Avg [mM]	StdErr	Avg [mM]	StdErr	Avg [mM]	StdErr	Avg [mM]	StdErr
WT	0.380	0.016	0.835	0.059	0.389	0.061	0.217	0.037
cp12	0.358	0.039	1.155	0.133	0.407	0.086	0.323	0.038
**Glutamic Acid**	Avg [mM]	StdErr	Avg [mM]	StdErr	Avg [mM]	StdErr	Avg [mM]	StdErr
WT	0.903	0.086	2.434	0.225	0.951	0.318	6.656	1.195
cp12	0.911	0.150	2.873	0.386	1.211	0.461	4.026	0.674
**Glutamine**	Avg [mM]	StdErr	Avg [mM]	StdErr	Avg [mM]	StdErr	Avg [mM]	StdErr
WT	0.155	0.020	0.386	0.044	0.131	0.025	0.267	0.018
cp12	0.171	0.015	0.981	0.042	0.137	0.029	0.240	0.022
**Glycine**	Avg [mM]	StdErr	Avg [mM]	StdErr	Avg [mM]	StdErr	Avg [mM]	StdErr
WT	0.102	0.021	0.091	0.001	0.073	0.016	0.081	0.004
cp12	0.093	0.031	0.118	0.009	0.091	0.015	0.073	0.005
**Histidine**	Avg [mM]	StdErr	Avg [mM]	StdErr	Avg [mM]	StdErr	Avg [mM]	StdErr
WT	0.009	0.004	0.017	0.001	0.016	0.006	0.011	0.002
cp12	0.016	0.011	0.024	0.002	0.013	0.004	0.008	0.001
**Isoleucine**	Avg [mM]	StdErr	Avg [mM]	StdErr	Avg [mM]	StdErr	Avg [mM]	StdErr

WT	0.010	0.001	0.009	0.001	0.022	0.004	0.010	0.001
cp12	0.014	0.003	0.015	0.001	0.015	0.001	0.010	0.001
**Leucine**	Avg [mM]	StdErr	Avg [mM]	StdErr	Avg [mM]	StdErr	Avg [mM]	StdErr
WT	0.013	0.001	0.014	0.000	0.020	0.005	0.017	0.001
cp12	0.016	0.004	0.017	0.001	0.016	0.003	0.016	0.000
**Lysine**	Avg [mM]	StdErr	Avg [mM]	StdErr	Avg [mM]	StdErr	Avg [mM]	StdErr
WT	0.154	0.022	0.381	0.043	0.131	0.020	0.268	0.018
cp12	0.171	0.016	1.001	0.057	0.139	0.025	0.241	0.022
**Methionine**	Avg [mM]	StdErr	Avg [mM]	StdErr	Avg [mM]	StdErr	Avg [mM]	StdErr
WT	0.009	0.001	0.009	0.000	0.007	0.001	0.005	0.000
cp12	0.010	0.001	0.013	0.001	0.007	0.000	0.007	0.001
**Phenylalanine**	Avg [mM]	StdErr	Avg [mM]	StdErr	Avg [mM]	StdErr	Avg [mM]	StdErr
WT	0.012	0.001	0.011	0.000	0.018	0.004	0.013	0.001
cp12	0.018	0.002	0.015	0.001	0.014	0.001	0.012	0.001
**Proline**	Avg [mM]	StdErr	Avg [mM]	StdErr	Avg [mM]	StdErr	Avg [mM]	StdErr
WT	0.014	0.004	0.122	0.035	0.025	0.004	0.018	0.003
cp12	0.016	0.005	0.219	0.076	0.022	0.005	0.020	0.001
**Serine**	Avg [mM]	StdErr	Avg [mM]	StdErr	Avg [mM]	StdErr	Avg [mM]	StdErr
WT	0.090	0.015	0.150	0.003	0.158	0.030	0.090	0.009
cp12	0.115	0.040	0.186	0.015	0.150	0.019	0.081	0.008
**Threonine**	Avg [mM]	StdErr	Avg [mM]	StdErr	Avg [mM]	StdErr	Avg [mM]	StdErr
WT	0.089	0.009	0.136	0.002	0.081	0.010	0.074	0.007
cp12	0.092	0.022	0.201	0.007	0.089	0.015	0.077	0.009
**Tryptophan**	Avg [mM]	StdErr	Avg [mM]	StdErr	Avg [mM]	StdErr	Avg [mM]	StdErr
WT	0.002	0.000	0.002	0.000	0.002	0.000	0.002	0.000

cp12	0.003	0.000	0.003	0.000	0.002	0.000	0.002	0.000
**Tyrosine**	Avg [mM]	StdErr	Avg [mM]	StdErr	Avg [mM]	StdErr	Avg [mM]	StdErr
WT	0.007	0.001	0.010	0.001	0.010	0.002	0.013	0.002
cp12	0.013	0.003	0.028	0.001	0.009	0.001	0.011	0.001
**Valine**	Avg [mM]	StdErr	Avg [mM]	StdErr	Avg [mM]	StdErr	Avg [mM]	StdErr
WT	0.020	0.003	0.031	0.002	0.033	0.009	0.022	0.002
cp12	0.023	0.007	0.037	0.006	0.027	0.005	0.023	0.001

**Others**	**HC steady state**		**LC 1h**		**LC 3h**		**LC steady state**	
**Ornithine**	Avg [mM]	StdErr	Avg [mM]	StdErr	Avg [mM]	StdErr	Avg [mM]	StdErr
WT	0.179	0.057	0.721	0.048	0.165	0.046	0.141	0.002
cp12	0.227	0.092	0.752	0.129	0.167	0.025	0.142	0.004
**Citrulline**	Avg [mM]	StdErr	Avg [mM]	StdErr	Avg [mM]	StdErr	Avg [mM]	StdErr
WT	0.059	0.010	0.218	0.018	0.016	0.005	0.150	0.035
cp12	0.043	0.022	0.319	0.040	0.024	0.005	0.172	0.030
